# Pro-survival function of MEF2 in cardiomyocytes is enhanced by *β*-blockers

**DOI:** 10.1038/cddiscovery.2015.19

**Published:** 2015-09-14

**Authors:** S Hashemi, J Salma, S Wales, JC McDermott

**Affiliations:** 1 Department of Biology, York University, Toronto, Canada; 2 Muscle Health Research Centre (MHRC), York University, Toronto, Canada; 3 Centre for Research in Biomolecular Interactions (CRBI), York University, Toronto, Canada; 4 Centre for Research in Mass Spectrometry (CRMS), York University, Toronto, Canada

## Abstract

*β*1-Adrenergic receptor (*β*1-AR) stimulation increases apoptosis in cardiomyocytes through activation of cAMP/protein kinase A (PKA) signaling. The myocyte enhancer factor 2 (MEF2) proteins function as important regulators of myocardial gene expression. Previously, we reported that PKA signaling directly represses MEF2 activity. We determined whether (a) MEF2 has a pro-survival function in cardiomyocytes, and (b) whether *β*-adrenergic/PKA signaling modulates MEF2 function in cardiomyocytes. Initially, we observed that siRNA-mediated gene silencing of MEF2 induces cardiomyocyte apoptosis as indicated by flow cytometry. *β*1-AR activation by isoproterenol represses MEF2 activity and promotes apoptosis in cultured neonatal cardiomyocytes. Importantly, *β*1-AR mediated apoptosis was abrogated in cardiomyocytes expressing a PKA-resistant form of MEF2D (S121/190A). We also observed that a *β*1-blocker, Atenolol, antagonizes isoproterenol-induced apoptosis while concomitantly enhancing MEF2 transcriptional activity. *β*-AR stimulation modulated MEF2 cellular localization in cardiomyocytes and this effect was reversed by *β*-blocker treatment. Furthermore, Kruppel-like factor 6, a MEF2 target gene in the heart, functions as a downstream pro-survival factor in cardiomyocytes. Collectively, these data indicate that (a) MEF2 has an important pro-survival role in cardiomyocytes, and (b) *β*-adrenergic signaling antagonizes the pro-survival function of MEF2 in cardiomyocytes and *β*-blockers promote it. These observations have important clinical implications that may contribute to novel strategies for preventing cardiomyocyte apoptosis associated with heart pathology.

## Introduction

Morbidity and mortality associated with heart disease remain a prevalent worldwide health concern.^1^ In the diseased heart, the lack of capacity for tissue regeneration after injury contributes to diminished cardiac function and heart health. At the cellular level, irreversible loss of cardiac myocytes due to programmed cell death contributes to pathological ventricular remodeling and progression to heart failure.^2,3^ Therefore, understanding the molecular genetic pathways that induce and also prevent myocardial cell apoptosis has potentially profound implications for understanding heart pathology and also therapeutic interventions for heart disease.^2,4^

*β*-Adrenergic receptor (*β*-AR) antagonists, or *β*-blockers, are a class of highly effective front-line drugs for heart disease that, at the molecular level, block norepinephrine and epinephrine from binding to the *β*-ARs. *β*-Blockers primarily block *β*1 and *β*2 receptors and their efficacy in the heart is related to their capacity to influence both chronotropy and inotropy by reducing both heart rate and the force of myocardial contraction. Thus, this reduction in heart work by *β*-blockers has been effectively used to ameliorate hypertrophy and cardiac dilation leading to heart failure.^5^ Interestingly, one reported benefit of chronic *β*-blocker treatment is in reducing myocyte death in patients with heart failure.^6,7^ Although activation of *β*1-AR, the predominant *β*-AR in the heart, has an important role in the regulation of normal heart function, prolonged activation of the *β*-adrenergic system in human heart disease or in experimental model systems results in dilated myopathy, cardiac fibrosis, cardiac myocyte apoptosis and heart failure.^8,9^ Previous studies have suggested that the cAMP/PKA pathway downstream of *β*1-AR induces cardiac myocyte apoptosis which is supressed by *β*-blocker therapy.^2,3^ However, the mechanism by which prolonged *β*-adrenergic activation compromises the survival of cardiac myocytes has not previously been elucidated.

Recently, we have reported that one of the myocyte enhancer factor 2 (MEF2) proteins (MEF2D), which is a key transcriptional regulator of cardiac gene expression, is directly targeted by PKA signaling.^10^ Although the role of MEF2 proteins in cardiovascular development and post-natal growth and hypertrophy has been extensively documented,^11–14^ there has previously been no clear link between MEF2 and cell survival pathways in the heart. MEF2s belong to the MADS (MCM1, agamous, deficiens, serum response factor) superfamily of sequence specific DNA-binding transcription factors. The N terminus of MEF2 proteins is highly conserved among all family members and consists of a 58-amino acid MADS domain and an immediately adjacent 28-amino acid MEF2 domain. These two domains collectively mediate dimerization, co-factor interactions and DNA binding to the consensus DNA sequence (C/T)TA(A/T)_4_TA(G/A) found in the regulatory region of most cardiac-specific genes.^15^ The two major isoforms of MEF2 in the adult heart are MEF2A and MEF2D, which form heterodimers.^16^ Although a pro-survival role of MEF2 has not been reported in striated muscle cells, a potent role of MEF2 in neuronal survival pathways has indeed been reported.^17^ Moreover, we recently documented that PKA activation leads to abrogation of MEF2 activity and pro-survival mechanisms in primary hippocampal neurons.^18^ This effect is mediated by a direct repressive effect of PKA phosphorylation of MEF2D at serines 121 and 190 leading to inhibition of its transactivation properties.^10^ In view of the potent role of both MEF2 and *β*-adrenergic signaling in the molecular control of heart structure and function, we explored a possible connection between *β*-adrenergic signaling and MEF2 in cardiomyocyte survival.

Here, we report, for the first time, that MEF2 has a pivotal pro-survival role in cardiomyocytes. Moreover, we observed that *β*-adrenergic activation directly antagonizes this MEF2 pro-survival role and *β*-adrenergic blockade restores this function, promoting cardiomyocyte survival. In addition, we document that the Kruppel-like factor 6 (KLF6) is an important downstream target of MEF2 in the cardiomyocyte survival pathway. These observations have important clinical implications for heart disease, firstly establishing that MEF2 has a key pro-survival role in the heart and secondly, documenting that *β*-adrenergic signaling intersects with this MEF2 survival pathway establishing a therapeutic node for intervention in cardiomyocyte apoptosis.

## Results

### MEF2 functions as a pro-survival factor in cardiac myocytes

**Figure 1:**
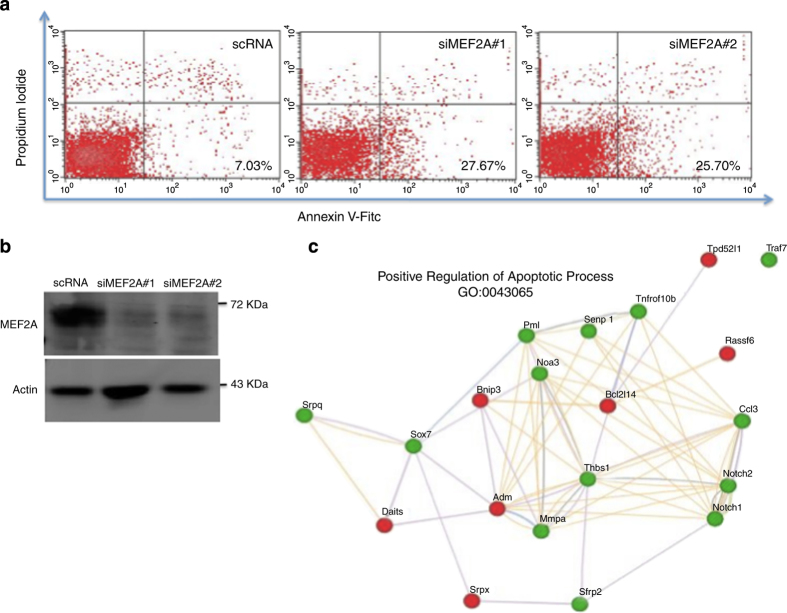
MEF2A knockdown induces apoptosis in cardiomyocytes. (**a**) Annexin V detection is upregulated in MEF2A-depleted cardiomyocytes. Primary cardiomyocytes were transfected with two independent MEF2A siRNAs or a control scRNA. Cells were stained with annexin V-FITC and PI using annexin V-FITC apoptosis detection kit 48 h after transfection. Apoptosis was measured using flow cytometry analysis (FACS analyzer). (**b**) siRNA-mediated gene silencing reduces MEF2A protein. Equal amounts of total protein were used for western blot analysis. The levels of the indicated proteins were assessed by a standard immunoblotting technique using specific primary antibodies for each as indicated. (**c**) RNA-seq analysis of MEF2A knockdown in cardiomyocytes. siMEF2A#1 or a control scRNA were transfected in cardiomyocytes in duplicate and prepared for RNA-seq analysis. The gene ontology (GO) term, positive regulation of apoptotic process, was observed. The 20 genes within this category with the lowest *P*-value are shown in a network created by the program GeneMania. Green and red nodes indicate down- and upregulated genes, respectively, whereas connections between nodes are as follows: purple, co-expression; orange, predicted; blue, co-localization; green, shared protein domains; gray, other.

MEF2 has previously been implicated in cell survival in primary embryonal hippocampal neurons^18^ and in cerebellar granular neurons.^19^ Although it is well documented that MEF2 functions as a key regulator of cardiac myocyte differentiation,^20,21^ its role, as a possible survival factor has not been proven. Recently, we have identified some potentially novel aspects of MEF2 function in skeletal and cardiac muscle using high throughput genomic technologies such as ChIP-Exo and RNA-seq which based on gene ontology analysis, suggest a wider role for MEF2A than just cellular differentiation.^22^ Interestingly, MEF2A-null mice exhibit an increase in cell mortality in the post-natal period.^20^ Therefore, we sought to rigorously address the question of whether MEF2 has a direct role in cardiomyocyte survival. Initially, we used siRNA-mediated gene silencing to downregulate MEF2A expression in primary cardiac myocytes followed by flow cytometry analysis to detect apoptotic cells. The efficacy of MEF2A silencing was tested by western blot analysis of primary cardiomyocytes that were transfected with two independent MEF2A siRNAs and a control scrambled siRNA (scRNA). As shown in Figure 1b, a robust reduction of MEF2A protein level was observed in cells expressing siMEF2A in contrast to cells expressing scRNA. To examine whether MEF2A silencing resulted in cardiomyocyte apoptosis, a combination of PI and annexin V-FITC fluorescence was used to determine necrosis and apoptosis by flow cytometry. An increase in the percentage of apoptotic cells with cardiomyocyte MEF2A silencing was observed as indicated in the lower right quadrant of the density plot (high Annexin V, low PI). Depletion of MEF2A enhanced the levels of cardiomyocyte apoptosis (27.67 and 25.70%) compared with the scRNA control condition (7.03%; Figure 1a). These data indicate that MEF2A has a pro-survival role in cardiomyocytes.

Next, we interrogated the role of MEF2A depletion in cardiomyocyte gene expression in RNA-seq. A full description and bioinformatics analysis of these RNA-seq data will be published elsewhere (Hashemi and Wales, in preparation). However, with respect to apoptosis, we observed a significant enrichment of the gene ontology term, ‘positive regulation of apoptotic process’ in the data set. Using GeneMania,^23,24^ the 20 genes with the lowest *P*-value were plotted to demonstrate the potential role MEF2A may have in regulating the expression of apoptosis-related genes. Several key apoptotic and tumor-suppressor genes are upregulated including Bcl2l14,^25^ Bnip3,^26,27^ Rassf6^28^ and Ddit4.^16^

### Acute *β*1-AR signaling represses MEF2 activity leading to cardiomyocyte apoptosis

**Figure 2:**
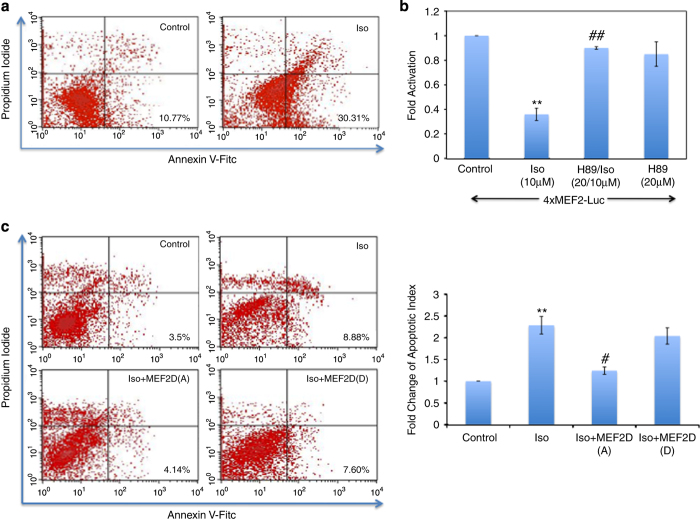
Activation of *β*1-AR induces cardiomyocytes apoptosis through the PKA pathway. (**a**) Primary cardiomyocytes were treated with Iso (10 *μ*M) for 48 h and then stained with annexin V-FITC and PI using annexin V-FITC apoptosis detection kit. Apoptosis was measured using flow cytometry analysis. (**b**) Cardiomyocytes were transfected with 4xMEF2-Luc reporter gene and treated with Iso (10 *μ*M) alone or in combination with PKA inhibitor (H89, 20 *μ*M). Luciferase activity was assessed using the respective reporter gene and normalized to *β*-galactosidase (*β*-gal) activity. Data are the mean±S.E. The quantification data is between the same batch with *n*=3, **P≤0.01 comparing Iso with control, ^##^*P*≤0.01 comparing H89/Iso to Iso. (**c**) PKA-resistant MEF2D rescues cardiomyocytes. Primary cardiomyocytes were transfected with empty vector or mutated forms of MEF2D S121/190A (MEF2D(A), neutralizing) and S121/190D (MEF2D(D), phospho-mimetic) and then treated with with Iso (10 *μ*M). Cells were prepared for FACS analysis as in **a**. *n*=3, **P≤0.01 Iso *versus* control, ^#^*P*≤0.05 Iso+MEF2D(A) *versus* Iso.

It has been documented that apoptotic cell death can be induced by *β*1-AR activation and is cAMP–PKA dependent.^2,3^ In skeletal muscle, we have previously shown that cAMP/PKA signaling is a potent repressor of MEF2D function and myogenic differentiation.^10^ As the *β*-adrenergic system is such an important regulator of physiological and pathological heart function, we sought to determine whether acute *β*1-AR activation might impinge on the survival function of MEF2. Initially, we assessed activation of *β*1-AR-mediated apoptosis in primary cardiomyocytes by flow cytometry. Estimation of necrosis and apoptosis were again determined by a combination of PI and annexin V-FITC fluorescence, respectively. Substantial increases in apoptotic cells (cells appearing in the lower right quadrant of the density plot) were observed with isoproterenol (Iso; 10 *μ*M) treatment (30.31%) when compared with control cells (10.77%; Figure 2a). To determine whether the mechanism leading to apoptosis is through cAMP–PKA pathway, we used a well-known pharmacological inhibitor of PKA, H89 (20 *μ*M) before treatment with Iso (10 *μ*M).

Subsequently, reporter gene analysis demonstrated a reduction of MEF2 activity in Iso-treated cells, which was reversed by H89 treatment (Figure 2b). MEF2A and MEF2D are the two major isoforms of MEF2 in post-natal hearts that form heterodimers.^16^ PKA was found to directly phosphorylate MEF2D in our previous work,^10^ which also showed that this repressive effect was transdominant over MEF2A activity when MEF2A was heterodimerized with MEF2D, as is the case in cardiomyocytes. PKA directly phosphorylates S121 and S190 on MEF2D and these sites were sufficient for repressive effects on skeletal muscle differentiation in response to cAMP signaling.^10^ To investigate this further, we determined whether a PKA-resistant MEF2D mutation (MEF2D S121/190A) could ameliorate *β*1-AR-PKA-mediated apoptosis in response to Iso. We noted a decrease in apoptotic cardiomyocytes when S121/190A was overexpressed with Iso (10 *μ*M; 4.14%), compared with Iso alone (8.88%) in cardiomyocytes (Figure 2c). Conversely, phophomimic form of MEF2D (S121/190D) did not rescue Iso-induced cardiomyocyte cell death (Figure 2c). These results demonstrate that a PKA-resistant MEF2D (S121/190A) protects cardiomyocytes from Iso-induced apoptosis.

### A *β*1-selective adrenergic receptor blocker (Atenolol) enhances MEF2 transcriptional activity in primary cardiomyocytes

**Figure 3:**
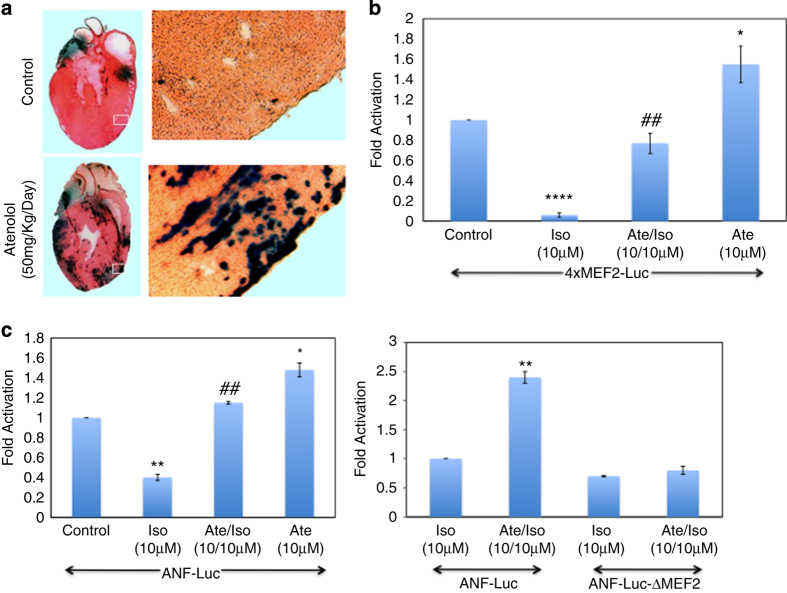
Ate enhances MEF2 transcriptional activity in cardiomyoytes. (**a**) Animal treatment with Ate *in vivo*. MEF2-LacZ transgenic mice were fed daily with Ate (50 mg/Kg per day) or water for 48 h. After treatment, mice were killed and hearts were fixed with 2% paraformaldehyde in PBS for 30 min. The samples were then incubated with X-Gal solution overnight and visualized for MEF2 activity. The dark blue stain indicates MEF2 activity. (**b**) Cardiomyocytes were transfected with 4xMEF2-Luc reporter gene and treated with Iso (10 *μ*M), Ate (10 *μ*M) alone or in combination. Luciferase activity was assessed using the respective reporter gene and normalized to *β*-galactosidase. Data are the mean±S.E. *n*=3, ****P≤0.0001 comparing Iso to control, **P≤0.01 comparing Ate/Iso to Iso, *P≤0.05 comparing Ate to control. (**c**) Ate enhances transactivation of the ANF promoter through MEF2. The effect of Iso (10 *μ*M), Ate (10 *μ*M) alone and in combination was assessed on ANF-Luc or an analog with the MEF2 site mutated (ANF-Luc ΔMEF2) in cardiomyocytes. Data are the mean±S.E. *n*=3, ****P≤0.0001 Iso *versus* control, ^##^ *P*≤0.01 Ate/Iso *versus* Iso, *P≤0.05 Ate *versus* control.

Given our data showing that *β*1-AR agonists activate PKA and induce apoptosis in cardiomyocytes, at least in part, by blocking the pro-survival role of MEF2A/D heterodimers, we next addressed the effects of *β*-blockers on MEF2 activity. As *β*-blocker therapy effectively is a first line treatment for most heart pathology,^3^ we hypothesized that its impact on MEF2-mediated pro-survival, in conjunction with the well-known effects of *β*-blockers on cardiac contractility could contribute to the protective effects of pharmacological blockade of *β*1-AR in the heart. To test this hypothesis, we initially used an *in vivo* model a MEF2-LacZ sensor mouse,^10,29–31^ were treated with either *β*-blockers (Atenolol (Ate)—50 mg/kg per day) or solvent (water) for 48 h. The heart tissue was then stained with X-Gal (5-bromo-4-chloro-3-indolyl-*β*-d-galactopyranoside) and visualized for MEF2 activity. The data indicated a substantial enhancement of MEF2 activity in relatively acute *β*-blocker treatment in the hearts, as illustrated by X-Gal staining (Figure 3a). To document these effects in a more controlled manner, primary cardiomyocytes *in vitro* were transfected with a 4xMEF2-Luciferase construct (a synthetic reporter gene containing four copies of the MEF2 *cis-*element in tandem) and were treated with Ate (10 *μ*M) alone and in combination with Iso (10 *μ*M) for 48 h before determination of MEF2 transcriptional activity. These data illustrate that Ate enhances MEF2 transcriptional activity (Figure 3b). To further corroborate this, the effect of *β*-blockers on a natural heart promoter (atrial natriuretic factor—ANF), a previously well-characterized MEF2 target gene,^32^ was analyzed. Standard reporter gene analysis was performed using the ANF-Luc reporter gene with Ate (10 *μ*M) and Iso (10 *μ*M) treatment. These experiments revealed that Iso (10 *μ*M) treatment repressed Ate-Luc activity and this effect was abrogated with Ate, consistent with the idea that *β*-agonists repress ANF promoter activity through the MEF2 *cis*-element (Figure 3c, left panel). This was confirmed by utilizing an ANF-Luc reporter gene construct in which the MEF2 site was mutated by substitution of the A/T rich core of the MEF2 site with a GGG tri-nucleotide, which completely abrogates MEF2 binding (ANF-Luc ΔMEF2) (Figure 3c, right panel). Collectively, these data indicate that cardiomyocyte MEF2 activity on synthetic and natural promoters is repressed by Iso treatment and de-repressed by Ate treatment. These data demonstrate a potent level of control of cardiomyocyte MEF2 activity by *β*-adrenergic signaling

### *β*-AR activation modulates MEF2D cellular localization in neonatal cardiomyocytes

**Figure 4:**
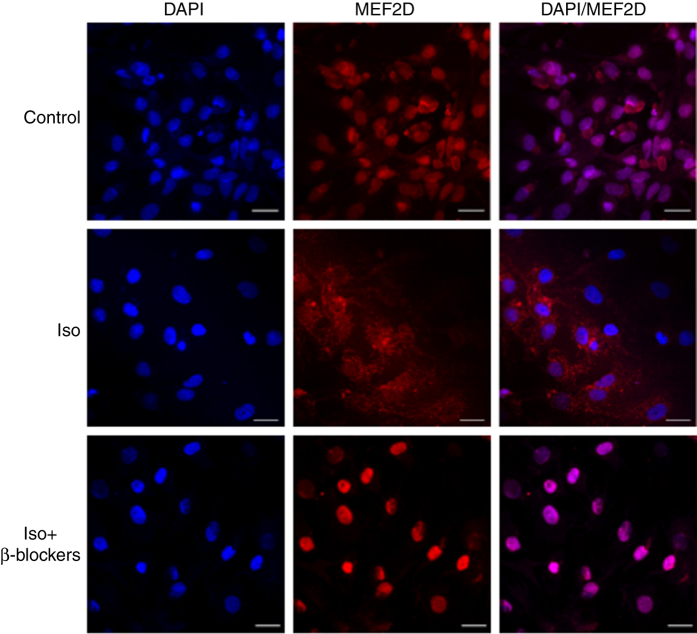
*β*-AR activation modulates cellular localization of MEF2D in cardiomyocytes. Primary cardiomyocytes were treated with solvent or Iso (10 *μ*M) alone and in combination with *β*-blockers Ate (10 *μ*M) and ICI118551 (1 *μ*M). After treatment, cells were fixed with 4% paraformaldehyde and immunofluorescence analysis was performed using a primary antibody to MEF2D (red). DAPI (4,6-diamidino-2-phenylindole) was used to identify nuclei (blue). The merged pictures demonstrate localization of MEF2D (red) in respect to Iso (10 *μ*M), *β*-blockers Ate (10 *μ*M) and ICI118551 (1 *μ*M) treatment, counterstained with DAPI. Scale bars, 20 *μ*m.

In attempting to determine the mechanism by which MEF2 activity is repressed by *β*-adrenergic signaling, we determined the subcellular localization of MEF2D in cardiomyocytes that were treated with Iso (10 *μ*M) alone and in combination with *β*-blockade Ate (10 *μ*M) and ICI118551 (1 *μ*M) using Immunofluorescence analysis. As shown in Figure 4 (top panel), MEF2D is mainly localized in the nucleus in solvent treated cells. However, we observed that in Iso-treated cells, MEF2D is mostly localized in the cytosol Figure 4 (middle panel). We further documented that with *β*-blocker treatment, MEF2D localization in the nucleus was restored Figure 4 (bottom panel). We observed a similar disruption of cellular localization patterns of MEF2A in cardiomyocytes treated with Iso alone and in combination with *β*-blockers (Supplementary Figure 1).

### KLF6 functions downstream of MEF2 as a pro-survival factor in cardiomyocytes

**Figure 5:**
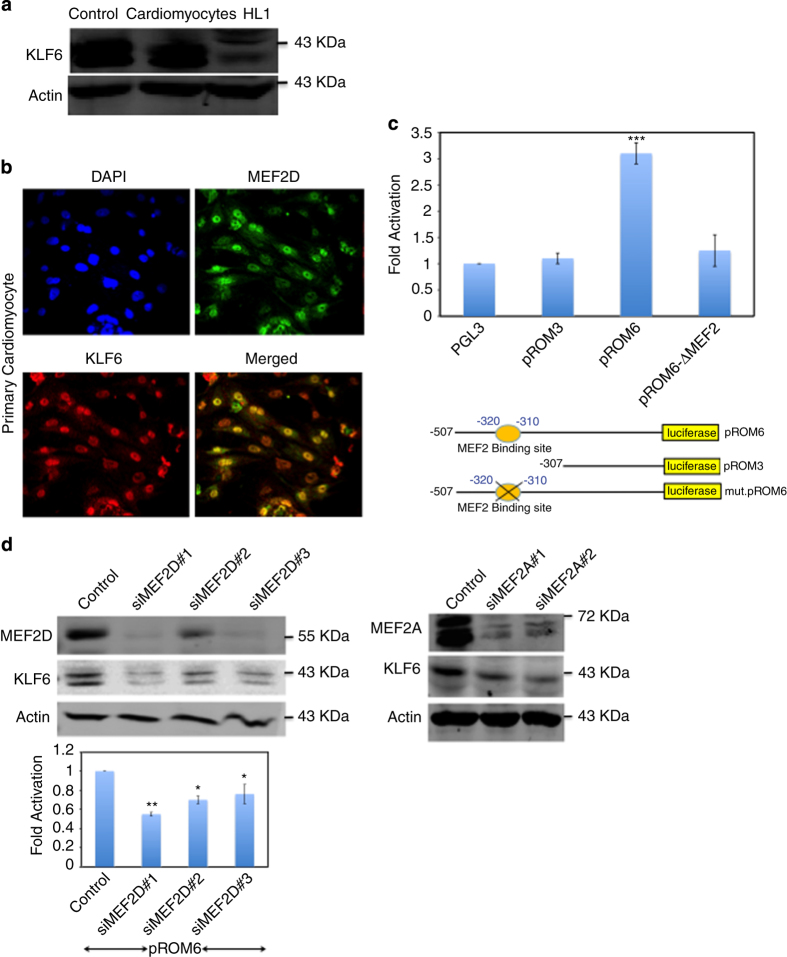
MEF2 regulates KLF6 expression in cardiomyocytes (**a**) KLF6 protein is expressed in cardiomyocytes. Cell lysates of primary cardiomyocytes and HL1 cells and C2C12 (as control) were prepared and equal amounts of total protein were used for western blot analysis. The levels of the indicated proteins were assessed by a standard immunoblotting technique using specific primary antibodies for each as indicated. (**b**) Cellular localization of MEF2D and KLF6 in cardiomyocytes. Primary cardiomyocytes were fixed with 4% paraformaldehyde. Double immunofluorescence labeling demonstrating KLF6 (red) and MEF2D (green) and DAPI (4,6-diamidino-2-phenylindole) was used to identify nuclei (blue). (**c**) MEF2-dependant induction of the KLF6 promoter in cardiomyocytes. Schematic illustrations of KLF6 reporter gene constructs used in reporter assays are indicated in the lower panel. All KLF6 promoter constructs were cloned into the pGL3-basic reporter vector (pGL3-KLF6-Luc). Primary cardiomyocytes were transfected with various constructs of the KLF6 promoter, pROM6, pROM3 and pROM6 with the MEF2 site mutated. Cell extracts were prepared and MEF2-mediated transcriptional activity was determined by luciferase and *β*-gal assays. *n*=3, ***P≤0.001 pROM6 *versus* pGL3. (**d**) KLF6 expression is reduced in MEF2 depleted cardiomyocytes. Primary cardiomyocytes were transfected with three independent MEF2D siRNAs (left panel) or two independent MEF2A siRNAs (right panel). Forty-eight hours after transfection equal amounts of total protein were used for western blot analysis. The levels of the indicated proteins were assessed by a standard immunoblotting technique using specific primary antibodies for each as indicated. KLF6 reporter gene expression is reduced in MEF2D depleted cells. Luciferase activity was assessed using KLF6 promoter driving luciferase (pROM6-Luc) and normalized to *β*-galactosidase. Data are the mean±S.E. (*n*=3), **P≤0.01, *P≤0.05 comparing to control.

We previously observed that KLF6 is a key MEF2D target gene in primary hippocampal neurons,^18^ and therefore sought to determine whether KLF6 might also function in cardiomyocytes. Endogenous expression of KLF6 was initially confirmed in primary neonatal cardiomyocytes and HL1 cells using western blot analysis (Figure 5a). Immunofluorescence analysis indicated expression of KLF6 in MEF2D positive primary cardiomyocytes. The data also indicate nuclear localization of both MEF2D (green) and KLF6 (red) in cardiomyocytes (Figure 5b). We also observed a similar cellular localization pattern of KLF6 and MEF2D in HL1 cells (Supplementary Figure 2). Firstly, we assessed MEF2-dependent regulation of the KLF6 promoter in cardiomyocytes. To do this we used a number of KLF6 promoter reporter gene constructs containing different fragments of the KLF6 promoter, pROM6 (contains MEF2 *cis*-element), pROM3 (which has no MEF2 site), mut.pROM6 (contains MEF2-binding site mutation) and pGL3-basic empty vector which was used as a control (schematic in Figure 5c, lower panel). As shown in Figure 5c (upper panel), endogenous MEF2-induced pROM6 reporter transcriptional activity in contrast to pROM3, which lacks the MEF2-binding site. Furthermore endogenous MEF2 did not induce KLF6 reporter transcriptional activity when the MEF2 site is mutated, indicating that MEF2 is a transcriptional regulator of the KLF6 promoter.

To further test if KLF6 is a potential MEF2 target gene in cardiac myocytes, we used siRNA targeting to reduce MEF2D and -A expression and then assessed KLF6 protein expression. Cardiomyocytes were transfected with three independent MEF2D siRNAs and scRNA. MEF2D silencing resulted in a concomitant repression of KLF6 protein expression (Figure 5d, left upper panel) corresponding with a decrease in KLF6 promoter activation (pROM6; Figure 5d, lower panel). In addition, the reduction of KLF6 protein level was also observed in cells expressing siMEF2A in contrast to cells expressing the scRNA control (Figure 5d, right panel).

### KLF6 protects cardiomyocytes in *β*1-AR–PKA pathway

**Figure 6:**
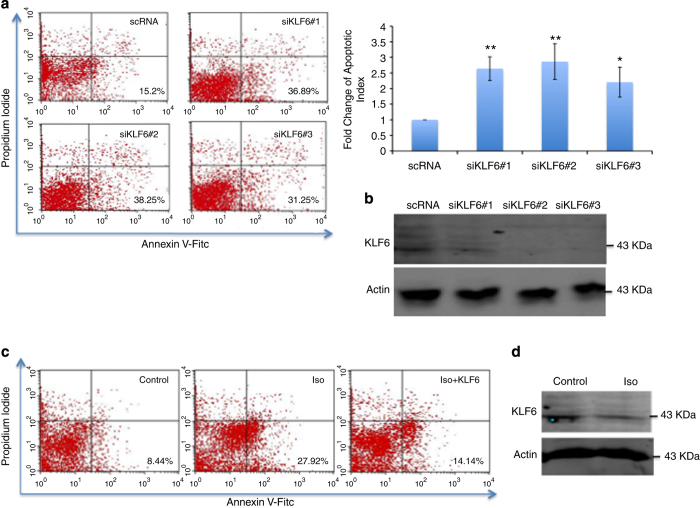
Role of KLF6 in cardiomyocyte survival. (**a**) siRNA-mediated depletion of KLF6 expression enhances apoptosis in primary cardiomyoytes. Primary cardiomyocytes were transfected with three independent KLF6 siRNAs and a control scRNA. Seventy-two hours after transfection, cells were stained with annexin V-FITC and PI using annexin V-FITC apoptosis detection kit. Cardiomyocytes apoptosis was measured using flow cytometry analysis (FACS analyzer). Changes in the number of apoptotic cells is indicated in a bar graph (left panel). *n*=3, **P≤0.01,*P≤0.05 comparing to control. (**b**) Equal amounts of total protein were used for western blot analysis to validate KLF6 knockdown. The levels of the indicated proteins were assessed by a standard immunoblotting technique using specific primary antibodies for each as indicated. (**c**) Exogenous KLF6 expression rescues cardiomyocytes apoptosis. Primary cardiomyocytes were transfected with KLF6 expression vector or a control vector and then treated with Iso (10 *μ*M). Cells were then prepared for FACS analysis. (**d**) Activation of *β*1-AR suppresses KLF6 protein expression. Primary cardiomyocytes were treated with Iso (10 *μ*M) or solvent. Cells were prepared for western blot analysis as in **b**.

In an attempt to assess the functional role of KLF6 in cardiomyocytes, we silenced its expression using siRNA technology. Neonatal cardiomyocytes were transfected with three independent KLF6-siRNAs or scRNA and cardiomyocyte apoptosis was measured by flow cytometry analysis as described above (Figure 6a, left panel). Quantitative analysis shows depletion of KLF6 expression resulted in 2- to 3-fold increase in cardiomyocyte apoptosis compared with the control condition (Figure 6a, right upper panel). Reduction of KLF6 protein was observed in cells expressing siKLF6 in contrast to the scRNA (Figure 6b).

**Figure 7:**
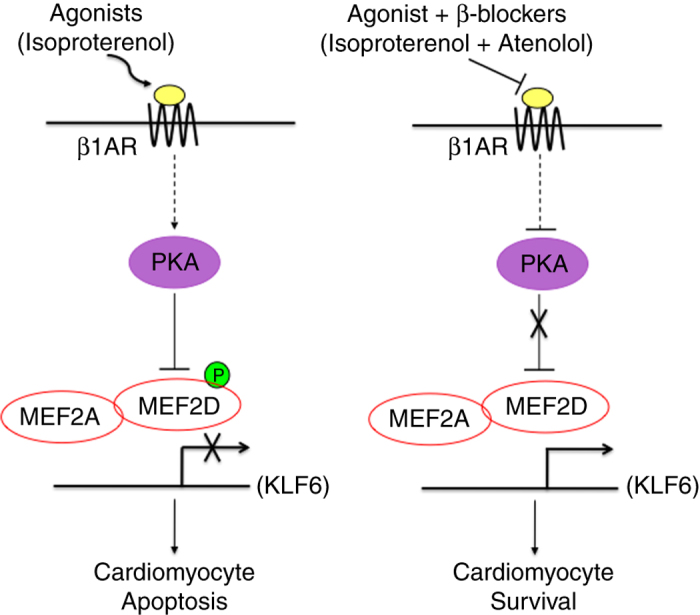
Summary of MEF2 regulation by *β*-adrenergic signaling in cardiomyocyte survival. On the left side, acute activation of *β*-adrenergic receptors invokes cAMP-mediated PKA activation in cardiomyocytes, resulting in suppression of MEF2 transcriptional activity by direct phosphorylation. Expression of pro-survival genes such as KLF6 is prevented resulting in enhanced cardiomyocyte death. On the right side, *β*-blockers, such as Atenolol, competitively inhibit the activation of the *β*-adrenergic receptors by agonists resulting in enhanced MEF2 activity, thereby promoting cardiomyocyte survival.

Interestingly, we also observed that exogenous overexpression of KLF6 can reduce the amount of cell death provoked by Iso treatment (Figure 6c). To examine whether the expression of KLF6 is targeted when MEF2 activity is repressed by *β*1-AR–PKA signaling, cardiomyocytes were treated with Iso (10 *μ*M) followed by western blot analysis of KLF6. As shown in Figure 6d, KLF6 protein expression level was suppressed by activation of *β*1-AR–PKA signaling. These data indicate that reduction in KLF6 expression by *β*1-AR activation occurs through MEF2 inhibition. Collectively, these data summarized in Figure 7, suggest that KLF6 functions downstream of MEF2 in a cardiomyocyte survival pathway.

## Discussion

Here, we present several lines of evidence documenting that MEF2 activity has an anti-apoptotic, pro-survival role in cardiac myocytes. Moreover, this pro-survival activity is antagonized by *β*-adrenergic signaling and, importantly, enhanced by *β*-adrenergic blockade (Figure 7). In view of the profound effects of myocyte loss in heart pathology, it is perhaps appropriate to consider possible contexts occurring in the cardiovascular system in which these observations have potentially important implications. One prominent context exists immediately after myocardial infarction, when the survival of cardiomyocytes in the myocardium is known to be severely compromised and also MEF2 activity is repressed by hyperactivation of *β*-adrenergic signaling and subsequent PKA-mediated phosphorylation. Our data indicate that *β*-blockade immediately post myocardial infarction could minimize cell death by promoting the cell survival mechanisms invoked by MEF2 and its downstream effectors.

There are other conditions of acute hyper-adrenergic activation in humans, apart from myocardial infarctions, that have been linked with heart pathology.^33^ In patients with stress cardiomyopathy a link between myocyte death and pronounced acute *β*-adrenergic activation has been postulated.^33^ Certainly it is clear that one seminal feature of progressive heart failure is an elevation in catecholamine levels that results in myocyte death and concomitant hypertrophy in surviving cells, ultimately contributing to a worsening of left ventricular function.^34–36^ Indeed, the general cardio-toxicity of high levels of catecholamines in the heart and more specifically the exact role of catecholamine mediated myocyte death is, to date, not well understood, although it has been reported that chronic hyperactivation of *β*-adrenoreceptors leads to a PKA-mediated phosphorylation of the Ryanodine receptor that results in calcium leakage from the sarcoplasmic reticulum, possibly invoking cell death mechanisms.^37^ Despite the link between cardiac pathology and elevated catecholamine levels documented in multiple,^33–37^ our knowledge of the mechanisms leading to cell death in these contexts is still incomplete. On the basis of our observations, we propose that acute *β*-adrenergic stimulation mediates inactivation of the pro-survival function of MEF2 in cardiac myocytes, thereby contributing to myocyte cell death and left ventricular dysfunction in a variety of pathologies ranging from myocardial infarction to stress induced cardiomyopathy. Moreover, acute *β*-adrenergic blockade restores MEF2 activity thus facilitating cardiac myocyte survival.

We report here that the expression of a substantial number of apoptotic network related genes is affected by experimental manipulation of MEF2 activity (Figure 1). For example, in our experiments several key apoptotic and tumor-suppressor genes are upregulated in response to MEF2A suppression by siRNA technology, suggesting that MEF2 ordinarily represses these pro-apoptotic genes, including Bcl2l14,^25^ Bnip3 (Bcl2- and 19KD-interacting protein-3),^26,27^ Rassf6^28^ and Ddit4 (DNA damage-inducible transcript 4). Bnip3-mediated cardiomyocyte apoptosis contributes to post-infarction left ventricular remodeling.^26,27^ Ddit4 is upregulated in response to ischemia/hypoxia-induced damage.^38^ In addition, many genes that are broadly described as ‘cardio-protective’ were downregulated by MEF2A suppression including, notably, Notch 1; Thbs1 (thrombospondin 1) and NOS3 (nitric oxide synthase 3). It was previously shown that Notch 1 signaling reduces cardiomyocyte apoptosis in ischemic post-conditioning.^39,40^ Thbs1 contributes to healing myocardial infarcts and also protects against cardiac remodeling by regulating TGF*β* signaling and promoting matrix preservation.^41,42^ Exogenous NOS3 expression in myocardium protects the heart from arrhythmia.^43^ Collectively, in general terms, it seems that loss of MEF2 function results in hyperactivation of genes involved in apoptotic induction and cell death while concomitantly leading to a reduction in the levels of genes involved in myocyte survival. The compound effects of these global gene expression changes for cardiac myocyte survival are clearly emphatic and of important clinical concern. Of note here is that in three independent large scale studies, the efficacy of *β*-adrenergic blockade in heart failure patients was reproducible and resulted in an approximate reduction by a third in the risk of death.^44^ A statistic that is unparalleled by any other drug used to treat heart failure.^44^ On the basis of our studies, our tenet is that there are no doubt multiple mechanisms contributing to this favorable outcome, one of which might be enhanced cardiac myocyte survival mediated by MEF2-dependent gene expression.

Our observations, in combination with those of other groups, suggest the possibility of a general role for MEF2 in cell survival. In particular, MEF2 has been implicated in neuronal survival^18^ and, in the current study, protection from cardiomyocyte cell death. Whether MEF2 has a general pro-survival role in other tissues is currently unknown. So far, MEF2 has been implicated in the control of gene expression and differentiation in neurons, cardiac, skeletal and smooth muscle, T and B cells, adipocytes and osteoblasts.^45–51^ It will therefore be of interest to determine in these other MEF2-dependent cell types whether cell survival is an ancillary function of its activity apart from its better characterized role in cellular differentiation. Gene targeting studies indicate that MEF2A has a role in cardiac metabolism, including regulation of fatty acid oxidation in the heart and maintenance of mitochondrial function.^20^ Activation of caspase 9 and the role of mitochondria in catecholamine-induced apoptosis in cardiomyocytes has also been documented.^3^ In view of these reports, it is likely that MEF2 protects against the mitochondrial-induced cell death pathway.

In view of the seemingly central role had by MEF2 in the control of gene expression in the heart, signal pathway regulated control of MEF2 activity could offer a broad target for therapeutic intervention. Research to date has indicated that MEF2 is a conduit for several signaling pathways that are regulated by a variety of cellular signaling pathways. To date MEF2 activity has been shown to be directly modulated by p38 MAP kinase,^30,52^ ERK5/BMK1,^53^ PKA^10^ and CDK4^54^ kinases, which are themselves, controlled by a myriad of signaling pathways. Therefore, there are many nodes that constitute potential rheostats in regulating MEF2 activity in the heart by means other than *β*-adrenergic blockade. One distinct and immediately applicable possibility is through the use of HDAC inhibitors. Several studies have indicated that class II HDACs are the most potent physiological repressors of MEF2 activity.^55,56^ Since there are numerous well-characterized inhibitors of HDACs, some of which are in clinical trials, their utility to de-repress MEF2 activity in come cases may be worth consideration. Thus, signaling pathway diversity may provide considerable flexibility in targeting MEF2 under conditions in which it’s activity is necessary or advantageous, such as during acute impending cardiomyocyte cell death, as well as under conditions when its activity needs to be restricted, such as under chronic conditions leading to cardiomyocyte hypertrophy in which MEF2 contributes to cell enlargement by activation of fetal structural genes.

### Summary

Our knowledge of the mechanisms controlling cardiac myocyte cell death is still quite incomplete. On the basis of our observations, we propose that the transcriptional regulator MEF2 fulfills a critical pro-survival function in cardiac myocytes having important implications for our understanding and therapeutic targeting of myocyte cell death and left ventricular pathology.

## Materials and methods

### Cell culture

Primary neonatal rat cardiomyocytes were prepared from 1- to 3-day old Sprague Dawley rats using the Neonatal Cardiomyocyte Isolation System (Worthington Biochemical Corp, Lakewood, NJ, USA). Briefly, whole hearts were dissociated with trypsin (Promega, Madison, WI, USA) and collagenase (Worthington Biochemical Corp). The cells were re-suspended in Dulbecco's modified Eagle's medium F12 (Gibco, Burlington, ON, Canada) supplemented with 10% fetal bovine serum, 1% penicillin/streptomycin and 50 mg/l gentamycin sulfate (Invitrogen, Burlington, ON, Canada). The isolated cells were plated for 60 min in 37 °C humidified incubator with a 5% CO_2_ in air, allowing differential attachment of non-myocardial cells. The cardiomyocytes were counted and transferred to gelatin-coated plates. The day after, medium was removed and replaced by fresh medium. For pharmacological treatments, cells were serum starved for the indicated time and replenished with fresh medium every 24 h.

### Atenolol administration *in vivo*

MEF2-LacZ transgenic sensor mice, reported previously,^10,29–31^ were used in this study. Two groups of male mice (*n*=5/each group) at 6–7 weeks were used. *β*-Blockers were administered through drinking water (Atenolol; 50 mg/kg per day) or solvent (5 ml water) for 2 days. Mice were sacrificed by cervical dislocation. The apex of each heart was fixed with 2% paraformaldehyde in PBS for 30 min. After being washed three times with PBS, the samples were incubated at 37 °C with X-Gal staining solution (5 mM ferrocyanide, 5 mM ferricyanide, 2 mM MgCl_2_, and 1 mg/ml X-Gal) to visualize *β*-Gal positive cells. Samples were examined using bright field microscopy.

### Reagents and antibodies

Rabbit polyclonal MEF2A antibody was produced with the assistance of the York University (Toronto, ON, Canada) Animal Care Facility. MEF2D (BD Biosciences, Mississauga, ON, Canada, 610775), KLF6 (Santa Cruz, Dallas, TX, USA, R-173 and E-10), Actin (Santa Cruz, sc-1616), IRDye 680RD goat anti-rabbit (LI-COR, Bioscience, Lincoln, NE, USA) and IRDye 680RD goat anti-mouse (LI-COR, Bioscience) were used for Immunoblotting experiments. FITC- and TRITC-conjugated *α*-rabbit and *α*-mouse secondary antibodies and 4,6-diamidino-2-phenylindole (DAPI; D9542), H_2_O_2_ (H0904) and H89-dihydrochloride hydrate (B1427) were purchased from Sigma Aldrich (Toronto, ON, Canada). Atenolol (Sigma Aldrich, A7655), Isoproterenol hydrochloride (Sigma Aldrich, 1351005) and ICI 118551 hydrochloride (Abcam, Toronto, ON, Canada, ab1200808) were purchased for use in cell culture.

### Plasmids

The firefly luciferase reporter gene plasmid pGL3–4xMEF2-Luc was made from pGL3-MEF2-Luc with three additional copies of the MEF2 site inserted.^10^ ANF and ANF ΔMEF2 reporter constructs (consist of the firefly luciferace cDNA driven by 700 bp of rat ANF promoter sequence), provided by Dr. M. Nemer (Faculty of Medicine, University of Ottawa). The expression plasmid of pCMV-*β*-galactosidase has been described previously.^10^ The expression plasmid for pCIneo-KLF6 was a provided by Dr. S. Friedman (Mount Sinai School of Medicine, New York, NY, USA). KLF6 reporter constructs pROM6 and pROM3-Luc were provided by Dr. N.P. Koritschoner (Faculty of Bioquimica y Ciencias Biologicas, Universidad Nacional del Litoral, Santa Fe, Argentina). pROM6 reporter construct containing the mutated MEF2-binding site (pROM6 ΔMEF2) has been described previously.^18^

### siRNA-mediated gene silencing

Gene silencing of target genes was done using siRNA technology; siRNAs were purchased from Sigma Aldrich. siMEF2A#1 (SASI_Mm01_00120787), siMEF2A#2 (SASI_Mm01_00120788) and siMEF2D#1 (SASI_Rn01_00057714), siMEF2D#2 (SASI_Rn01_00057709), and siMEF2D#3 (SASI_Rn01_00057717) were used at 100 nM concentrations. We also used siKLF6#1 (SASI_Rn01_00082277), KLF6#2 (SASI_Rn01_00082278) and KLF6#2 (SASI_Rn01_00082280) at 100 nM concentrations.

### Transfections

Primary cardiomycoytes were transfected using Lipofectamine 2000 (Invitrogen) in a 1:2.5 mixture ratio of DNA to lipofectamine in Opti-MEM (Invitrogen) according to the manufacturer’s instructions. Cells were re-feed and allow for recovery for 24 h before harvesting, or pharmacological treatments. For siRNA experiments, Lipofectamine RNAiMAX (Invitrogen) was used according to the manufacturer’s instructions. Cells were harvested 48–72 h after transfection for western immunoblotting analysis to determine the efficacy of protein knockdown or flow cytometry analysis.

### Protein extraction

Protein samples were kept on ice during the entire procedure. Cells were washed twice with cold 1x Phosphate buffered saline (PBS). After aspirating the last PBS wash, 1.0 ml of cold 1x PBS was added to cells. Cells were then gently scraped with a rubber policeman and transferred to a new tube and then centrifuged for 5 min at 4 °C. After removing the PBS, the pellet was diluted with five times its volume in NP-40 lysis buffer (supplemented with 1 mM sodium orthovanadate, 1 mM PMSF and protease inhibitor cocktail (Sigma, P-8340). Cells were vortexed briefly every 10 min for a total of 40–45 min, and centrifuged at high speed and supernatant was transferred to a fresh tube. Protein concentrations were determined by Bradford assay (Bio-Rad) with bovine serum albumin as a standard.

### Immunoblots

Cells were harvested using an NP-40 lysis buffer (0.5% NP-40, 50 mM Tris, 150 mM NaCl, 10 mM sodium pyrophosphate, 2 mM ethylenediaminetetraacetic acid and 100 mM NaF), containing protease inhibitor cocktail (Sigma Aldrich), 1 mM phenylmethylsulfonyl fluoride (Sigma Aldrich) and 1 mM sodium orthovanadate (Bioshop, Burlington, ON, Canada). Total protein extracts (20–25 *μ*g) were diluted in sample buffer (SDS-polyacrylamide) containing *β*-mercaptoethanol, boiled for 5 min and electrophoretically resolved by 10% SDS-polyacrylamide gels, then transferred onto Immobilon-FL polyvinylidene difluoride membrane (Millipore, Fisher Scientific (distributor) Ottawa, ON, Canada). Non-specific binding sites were blocked with Odyssey Blocking Buffer (LI-COR). Membranes were incubated with primary antibodies overnight at 4 °C in Odyssey Blocking Buffer. Primary antibodies included MEF2A (1:250), MEF2D (1:500), KLF6 (1:500) and actin (1:1000). The blots were then incubated with the appropriate secondary antibodies IRDye 680RD goat anti-rabbit (1:5000) and IRDye 680RD goat anti-mouse (1:5000) for 2 h at room temperature and were imaged using LI-COR Odyssey System.

### Luciferase reporter gene analysis

Transcriptional assays were done using luciferase reporter plasmids. The cells were harvested for these assays using lysis buffer (20 mM Tris, (pH 7.4) and 0.1% Triton X-100). Lysate was briefly vortexed and centrifuged at maximum speed for 15 min at 4 °C. Enzymatic activity was measured in each sample on a luminometer using luciferase assay substrate (E2820, Promega) and values obtained were normalized to *β*-galactosidase activity and expressed as fold activation by arbitrarily setting the control condition to 1. All measurements were made in triplicate for at least three independent experiments.

### Statistical analysis

Data shown are mean±S.D. All data were verified in three independent experiments using the same batch of cardiomyocyte isolation. Independent two sample *t*-tests of all quantitative data were conducted, whereas a one-way analysis of variance followed by a Tukey HSD *post hoc* test was performed on experiments with greater than two conditions. *P*-values are indicated with respect to controls where appropriate and *P*<0.05 was considered statistically significant.

### Flow cytometry

Flow Cytometry analysis was performed as previously described^18^ using the annexin V-FITC apoptosis detection kit (APOAF, Sigma) following the manufacturer’s instructions. Primary cardiomyocytes were washed, briefly trypsinized, and then washed twice with cold PBS. Cells were pelleted by centrifugation and re-suspended in binding buffer followed by incubation with staining solution (annexin V-FITC and PI) for 15 min in the dark at 4 °C. The cells were re-suspended in binding buffer. Samples were analyzed immediately by flow cytometry fluorescence, respectively. Ten thousand cells from each sample were scanned and analyzed by FACS Calibur flow cytometry (BD, Mississauga, ON, Canada) using the standard configuration and parameters. Data acquisition and analysis was performed using the Cell-Quest software (BD). Necrosis and apoptosis were determined by PI (FL2) and annexin V-FITC (FL1) fluorescence, respectively.

### Immunofluorescence

Primary cardiomyocytes were fixed in 4% paraformaldehyde and permeabilized with 0.3% Triton X-100 in PBS for 5 min. Cells were blocked with 10% goat serum in PBS for 30 min at 37 °C and incubated overnight at 4 °C with primary antibodies MEF2D (1:100), MEF2A (1:100) and KLF6 (1:100) diluted in 1.5% goat serum. Cells were washed three times with PBS for 10 min and incubated with the appropriate TRITC- and FITC-conjugated secondary antibodies (1:500) in 1.5% goat serum for 2 h at room temparature. DAPI staining (Sigma Aldrich) was carried out during the last 15 min. Cells were washed three times with PBS and images were captured using a Zeiss LSM confocal microscope (Carl Zeiss, ON, Canada).

## Abbreviations

MEF2: myocyte enhancer factor 2
PKA: protein kinase A
KLF6: Kruppel-like factor 6
*β*1-AR: *β*1-adrenergic receptor
ANF: atrial natriuretic factor.
