# Supplementary material for: Pro-survival function of MEF2 in cardiomyocytes is enhanced by β-blockers
Source: Cell Death Discov. 2015 Sep 14;1:15019–. doi: 10.1038/cddiscovery.2015.19 (PMC4979494; doi:10.1038/cddiscovery.2015.19)
Supplement: Supplementary Figures [file cddiscovery201519-s1.doc]

**Supplemental material**

Includes: Figure: S1, S2 and Figure captions: S1, S2

**Supplemental Figures**

**Figure S1. β-AR activation modulates cellular localization of MEF2A in cardiomyocytes.** Primary cardiomyocytes were treated with solvent or Isoproterenol (Iso, 10 μM) alone and in combination with β-blockers Atenolol (Ate, 10 μM) and ICI118551 (1 μM). After treatment, cells were fixed with 4% paraformaldehyde and immunofluorescence analysis was performed using a primary antibody to MEF2A (red). DAPI (4,6-diamidino-2-phenylindole) was used to identify nuclei (blue). The merged pictures demonstrate localization of MEF2A (Red) in respect to Isoproterenol (Iso, 10 μM), β-blockers Atenolol (Ate, 10 μM) and ICI118551 (1 μM) treatment, counterstained with DAPI. Scale represents 20 μm.

**Figure S2. Cellular localization of MEF2D and KLF6 in HL1 cells.**  HL1 cells were fixed with 4% paraformaldehyde. Double immunofluorescence labeling demonstrating KLF6 (red) and MEF2D (green) and DAPI (4,6-diamidino-2-phenylindole) was used to identify nuclei (blue).
